# Effects of physical activity calorie expenditure (PACE) labeling: study design and baseline sample characteristics

**DOI:** 10.1186/s12889-017-4710-0

**Published:** 2017-09-12

**Authors:** Anthony J. Viera, Laura Tuttle, Emily Olsson, Julie Gras-Najjar, Ziya Gizlice, Derek Hales, Laura Linnan, Feng-Chang Lin, Seth M. Noar, Alice Ammerman

**Affiliations:** 1 Department of Family Medicine, University of North Carolina, 590 Manning Drive, Chapel Hill, NC 27599 USA; 2 Center for Health Promotion and Disease Prevention, University of North Carolina, Chapel Hill, USA; 3 Department of Health Behavior, University of North Carolina, Chapel Hill, USA; 4 Department of Biostatistics, University of North Carolina, Chapel Hill, USA; 5 School of Media & Journalism, University of North Carolina, Chapel Hill, USA; 6 Department of Nutrition, University of North Carolina, Chapel Hill, USA

**Keywords:** Calorie labeling, Physical activity, Obesity prevention policy

## Abstract

**Background:**

Obesity and physical inactivity are responsible for more than 365,000 deaths per year and contribute substantially to rising healthcare costs in the US, making clear the need for effective public health interventions. Calorie labeling on menus has been implemented to guide consumer ordering behaviors, but effects on calories purchased has been minimal.

**Methods:**

In this project, we tested the effect of physical activity calorie expenditure (PACE) food labels on actual point-of-decision food purchasing behavior as well as physical activity. Using a two-group interrupted time series cohort study design in three worksite cafeterias, one cafeteria was assigned to the intervention condition, and the other two served as controls. Calories from food purchased in the cafeteria were assessed by photographs of meals (accompanied by notes made on-site) using a standardized calorie database and portion size-estimation protocol. Primary outcomes will be average calories purchased and minutes of moderate to vigorous physical activity (MVPA) by individuals in the cohorts. We will compare pre-post changes in study outcomes between study groups using piecewise generalized linear mixed model regressions (segmented regressions) with a single change point in our interrupted time-series study. The results of this project will provide evidence of the effectiveness of worksite cafeteria menu labeling, which could potentially inform policy intervention approaches.

**Discussion:**

Labels that convey information in a more readily understandable manner may be more effective at motivating behavior change. Strengths of this study include its cohort design and its robust data capture methods using food photographs and accelerometry.

## Background

Innovative policies are needed to help curb the obesity epidemic. One policy intervention is the requirement included in the 2010 Patient Protection and Affordable Healthcare Act (ACA) that restaurants post nutrition information on their menus. This requirement expanded upon the 1994 Nutrition Label and Education Act that required standardized nutrition labels on packaged foods. Under the new ACA menu labeling requirement, restaurants with 20 or more locations nationally are required to display “clear and conspicuous” calorie information for the food on their menus and menu boards. Since food away from home is a significant part of the American diet, providing consumers with information on the calorie content of this food could theoretically lead them to make more informed food choices, potentially encouraging lower calorie purchases. However, studies on the effects of this type of menu labeling on food purchasing and actual calorie consumption are conflicting [1–7]. The totality of the current evidence suggests that calorie labeling does not have the intended effect of decreasing calories purchased or consumed [7].

In a nationally representative sample, a quarter of Americans reported they would like to see physical activity equivalents provided with calorie information [8]. A few studies have examined the potential effect of physical activity energy equivalent food labeling schemes [9, 10]. We developed the Physical Activity Calorie Expenditure label based on mixed-methods preliminary research [10, 11]. Through a series of focus groups, we assessed comprehension of a PACE labeling scheme and refined the format of the initial label through an iterative process [11]. Then, in a hypothetical scenario study, 802 respondents were randomized to one of four menu label types: no additional information, calories only, calories plus average minutes of walking, or calories plus average miles of walking to burn the calories in the food item [10]. Respondents shown the calories plus miles ordered an average of 194 fewer calories compared to no label and 101 fewer compared to those shown the calories-only label [10].

The purpose of this paper is to describe the design of the PACE Study in which we moved from testing potential effects in a hypothetical context to testing the effects of calorie expenditure labeling in a “real-world” setting. The PACE Study examined not only effects of physical activity calorie expenditure labeling on calories purchased, but also on actual levels of physical activity.

## Methods

### Study overview

**Fig. 1:**
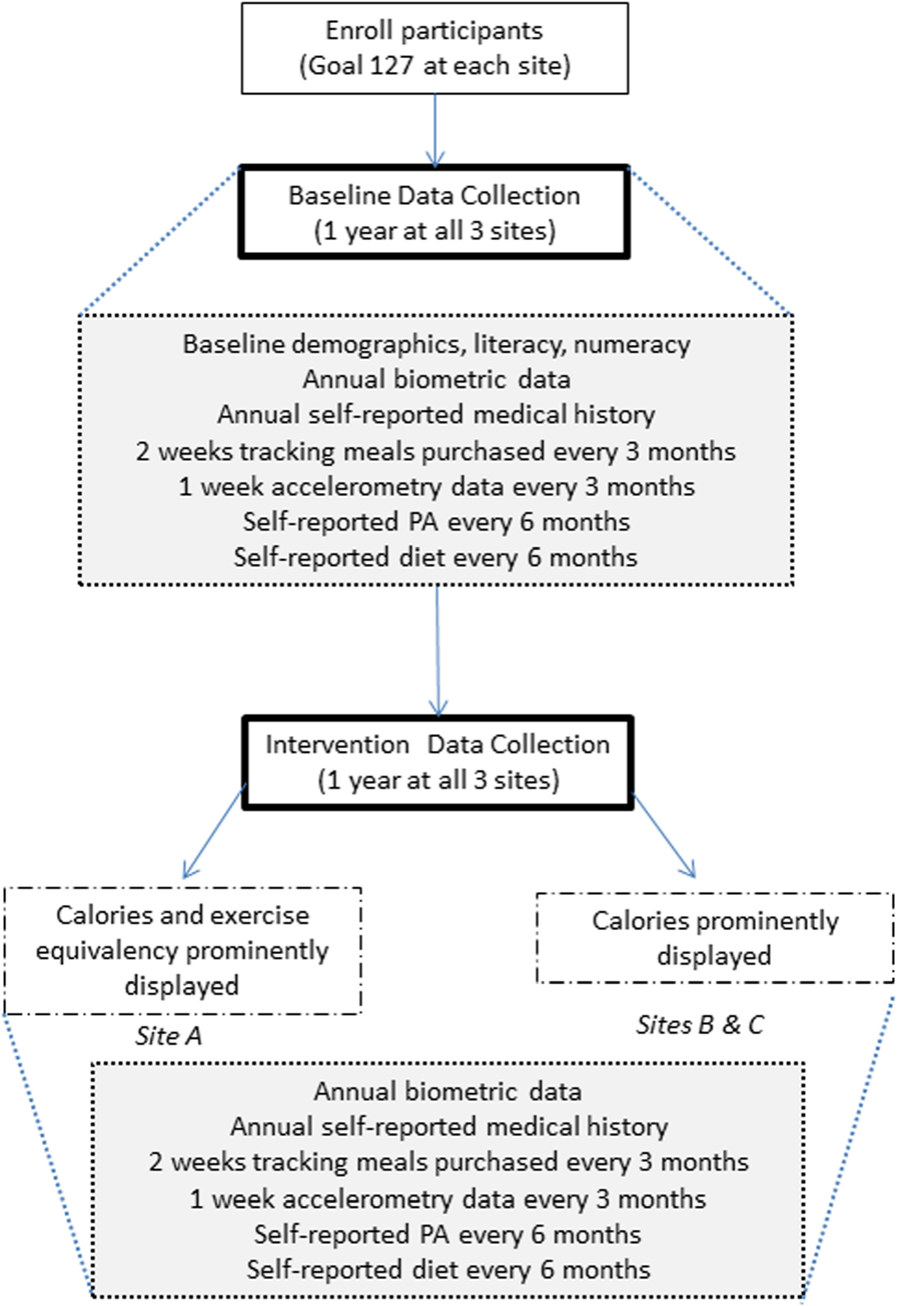
Overall study design

In partnership with Blue Cross and Blue Shield of North Carolina (BCBSNC), we tested the effects of PACE labels compared to calorie-only labels in three worksite campus cafeterias serving over 3600 employees. We enrolled participants from each worksite who eat lunch at the campus cafeterias and followed them for 24 months (Fig. 1). After a one-year pre-intervention (“baseline”) data collection period in all three cafeterias, one cafeteria had its food items labeled with PACE labels, and the other two cafeterias had their food items prominently displayed with calorie-only labels. The primary outcome will be changes in calories purchased by individuals enrolled in two cohorts from baseline to intervention year. As secondary measures, calories purchased were also assessed in aggregate (campus level) through purchase logs. Secondary outcomes will include changes in physical activity minutes, body mass index, blood pressure, glucose level, and cholesterol level.

### Participant recruitment and eligibility

We recruited participants through a combination of passive and active recruitment methods. Flyers (paper and electronic) advertising the study were placed throughout the BCBSNC campuses and on digital monitors. Additionally, study coordinators actively recruited participants in the worksite cafeterias by setting up an informational table for employees to visit and to learn more about or sign up for the study. Cohort participant enrollment continued on a rolling basis throughout the baseline year to help compensate for attrition. To meet inclusion criteria, a participant needed to (1) be a BCBSNC employee or contractor, and (2) eat lunch or be willing to eat lunch in the BCBSNC cafeteria at least 3 times per week.

### Enrollment visit

Enrollment visits were scheduled individually with the participants and a study coordinator in a private room on the BCBSNC campus. Study coordinators explained the details of the study and obtained the participants’ informed consent and HIPAA waiver. All participants were then asked to complete questionnaires including self-reported demographic items, medical and dietary history, and physical activity assessment forms on an electronic tablet [12, 13]. They also completed health literacy (Newest Vital Sign) and numeracy (3-item numeracy assessment) assessments [14, 15].

Participants were provided detailed information regarding the expectations of their participation and the timeline for data collection. To minimize participant awareness of the intervention differences between cafeterias, coordinators referred to the study by its working title, “Capturing Health Options at Work (CHOW)”, and participants were informed that the purpose of the study was to learn more about decisions made when choosing food away from home in a setting like a workplace cafeteria.

### Primary outcome measure

**Fig. 2:**
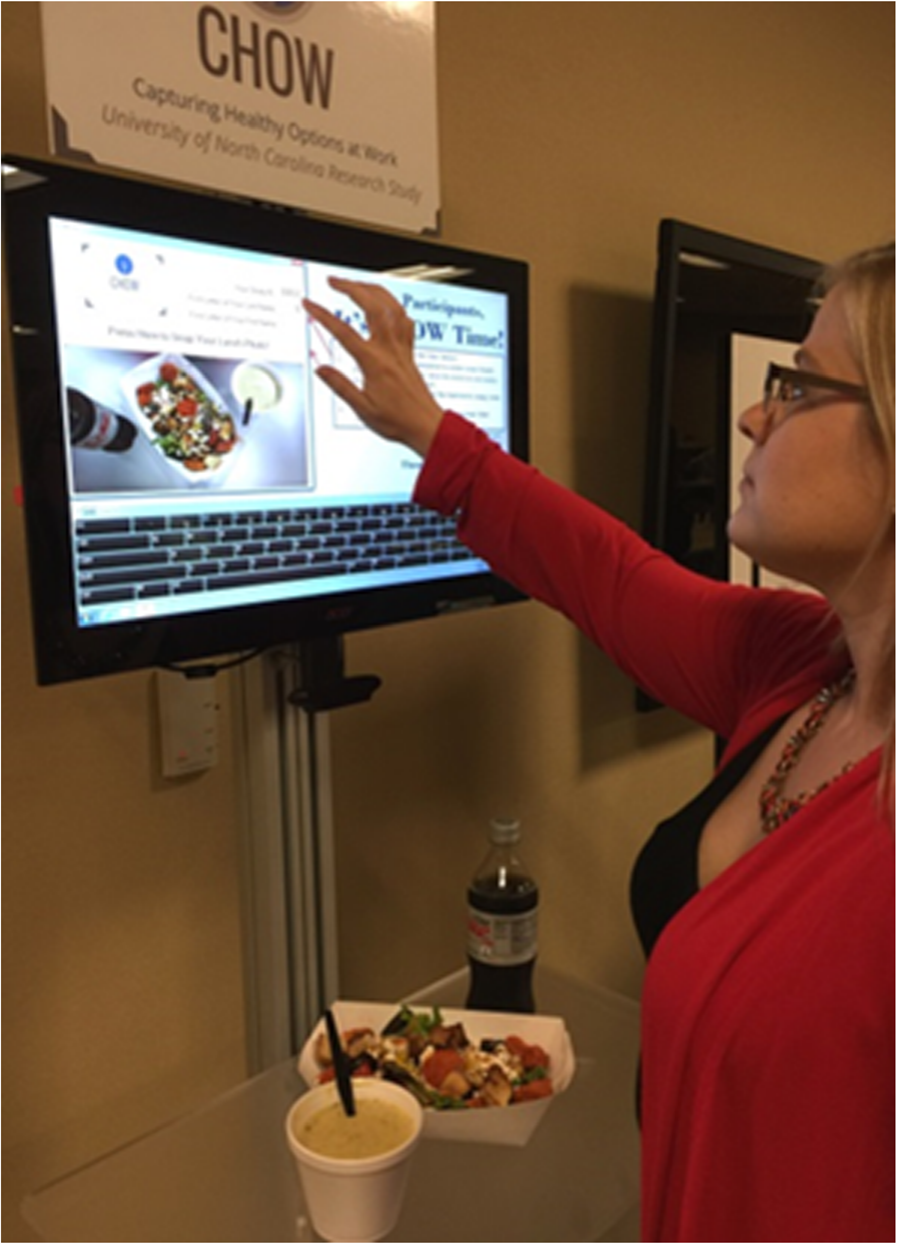
SnapFood™ photo capture system

In order to collect detailed information on the lunch purchases made by the participants, we developed SnapFood™, a photo capture system. SnapFood™ consists of a freestanding unit with a touchscreen monitor and camera positioned above a tray shelf (Fig. 2) which allows participants to submit their lunch photos quickly and objectively. The lunch photo was saved with the participants’ study identification number, initials, date and site location. The photo was automatically sent to a secure server at the University of North Carolina for analysis and storage. Study coordinators were present during data collection to assist participants and recorded any details that may not have been evident from the photograph (e.g., soup or drink contents, dressings, condiments). We designated the lunch photo data collection periods as “CHOW Time.”

**Fig. 3:**
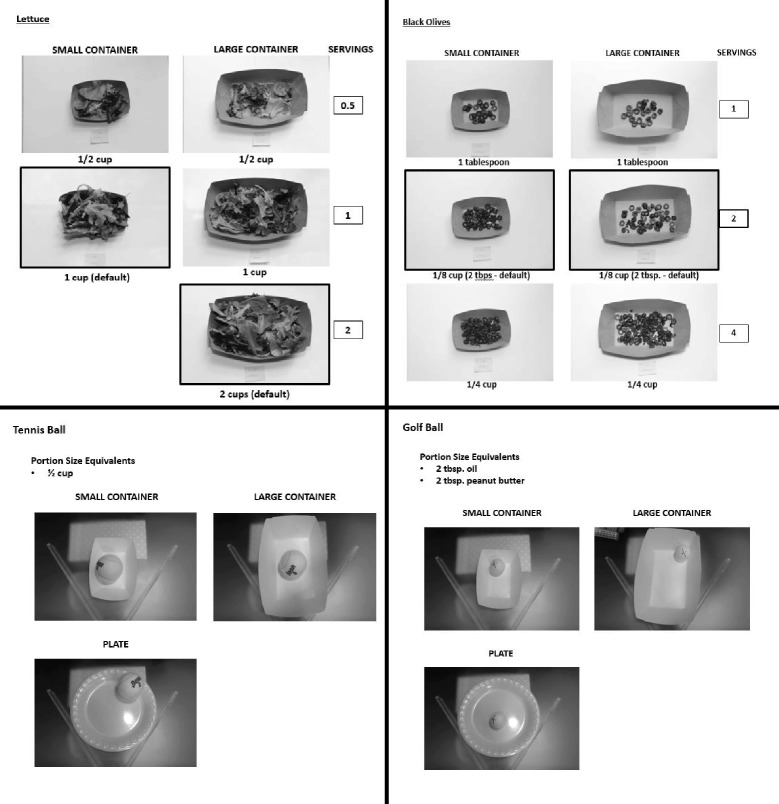
Example section from food atlas

During each “CHOW Time,” study coordinators analyzed the lunch photos and entered calorie information into a database. Item description, item code, item calories and number of servings were entered for each food item included in each lunch photograph. For any items requiring estimation of portion sizes (e.g., self-serve items or self-built salads), study coordinators used a food atlas (developed for this study, example shown in Fig. 3) along with notes documented during data collection to estimate portion sizes and determine caloric values. Coordinators underwent a series of reliability tests prior to actual data collection. Lunch photos were collected for 2 weeks at each of the three sites, on a quarterly basis during the 24-month study period.

### Other measures

Cohort participants were asked to wear an accelerometer (Actigraph wGT3X-BT) to collect physical activity data for one week at two different time points in each the baseline year and the intervention year. While wearing the activity monitor, participants received instructions to record their wake and sleep times as well as details regarding any activities not sufficiently captured by the monitor during wear (e.g., yoga, weightlifting). Accelerometry data were used to assess minutes engaged in moderate and vigorous physical activity (MVPA) as well as to categorize participants into physical activity levels.

During each “CHOW Time” lunch photo collection period, participants were also asked to complete questionnaires that were administered electronically (via cold fusion application developed for this study), either on a tablet device in the cafeteria or remotely on participants’ own devices. If any problems with electronic questionnaires arose, paper copies of questionnaires were available for completion by participants. Biometric data via health screenings are collected by BCBSNC for eligible employees (temporary and contract employees are not eligible for health screening) on an annual basis (voluntarily; not all cohort participants will have data collected). Biometric data include height, weight, body mass index, blood pressure, total cholesterol, high density lipoprotein (HDL), low density lipoprotein (LDL), triglycerides, and fasting blood glucose. BCBSNC cafeteria managers provided periodic reports of all foods purchased in each cafeteria.

### Labeling intervention

Following completion of the year of baseline data collection, new calorie labels were placed at each of the three study sites. Cafeteria A received PACE labels with calorie information and cafeterias B and C received calorie-only labels. As in our preliminary study [10], we determined PACE label values based on a 160-lb adult with an average calorie burn rate of 3.2 kcal/min while walking. By dividing the number of kcals in a given menu item by 3.2 kcal/min, we calculated the number of minutes needed to walk, which we then converted to miles, conservatively assuming a 30-min mile. Calorie-only labeling was chosen as the control given that such labeling is the currently recommended strategy. The labels (both PACE and calorie-only) were posted prominently at each corresponding food item. For food items that were not directly displayed, (food prepared to order at the grill and deli), lists of commonly purchased items were posted. For the self-serve salad bars, we posted lists of common items as well as representative salads showing the sum of calories from all ingredients included. Beverage cooler doors were labeled with lists of every beverage inside that specific door.

During weeks that we were not collecting data from cohort participants, individual labels were removed, while deli list, grill list, salad list and representative salads and comprehensive beverage lists were left in place.

**Fig. 4:**
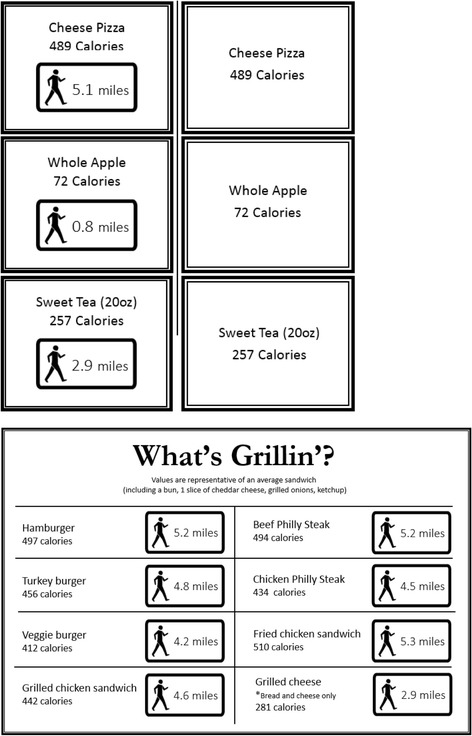
Example PACE and calorie-only labels and PACE food list

At the beginning of each quarterly data collection period, the study team re-posted brightly colored labels in a different color than used in the previous data collection period to draw attention to the labels. See Fig. 4 for example PACE and calorie-only labels and PACE lists.

### Statistical considerations and analysis plan

For our interrupted time series study design, we based sample size considerations for the primary outcome of change in purchase calories before versus after the intervention. For sample size calculations (116 participants per worksite), we assumed standard deviation of purchased meals is 350 cal and expected that calories plus PA labeling intervention would reduce purchase calories by 100 cal based on our preliminary study [10]. We will have four measures of average purchased calories at each quarter prior to the intervention and four after during 24-month study period. Thus, we will estimate and compare changes in calories purchased using a generalized linear mixed model (GLMM) that will include study groups, time, and study group by time interaction as fixed effects, each participant as random effect and a covariance structure that provides the best fit for the model. The null hypotheses are that the estimates for slopes before and after the intervention are the same for each study group.

Piecewise GLMMs (a.k.a. segmented regression analysis) will be fit with a single change point, after the fourth measurement time, corresponding to the initiation of the intervention to examine the changes (slopes) in these outcomes for this interrupted time series study to obtain estimates of slopes before and after introducing the calorie label interventions. The null hypothesis will test that the slope before the change point is the same as the slope afterwards. The slopes between groups after the intervention will be compared by using their respective error terms. We also realize that study groups could be different due to lack of randomization and will examine propensity scores and use them as weights for the GLM models. In addition, we will describe characteristics of all employees in each cafeteria and compare these characteristics to study participants to assess the representativeness of study sample. We will consider using sample characteristics that indicate under or over representation as covariates in our GLMMs and further conduct analyses to examine the effects of numeracy, literacy, education level, income, gender, age, and race by including study group x time x each of these variables in separate models.

## Results

### Baseline characteristics of population and participants

The majority of participants (*N* = 414) are female (77.8%). The sample is racially diverse with 45.4% white, 44.7% Black, and 5.3% identifying as Asian (Table 1). The demographics of the cohort largely reflect those of the entire employee population. Characteristics that differed between the participants across worksites included percent female and percent in various occupational roles.

**Table 1:** Characteristics of Cohort

	Entire Cohort (*n* = 414)	Cafeteria A (*n* = 160)	Cafeterias B&C (*n* = 254)
Mean age, years	42.2	41.1	42.9
Female, %	77.8	71.3	81.9
Race			
White	45.4	39.4	49.2
Black	44.7	46.9	43.3
Asian	5.3	8.1	3.5
Hispanic ethnicity, %	4.6	6.3	3.5
Education level %			
High school	12.6	11.9	13.0
College graduate	38.4	45.0	34.3
Master’s degree+	25.9	23.1	27.6
Current smoker, %	4.6	1.9	6.3
Adequate^a^ numeracy level	54.4	59.4	51.2
Health literacy items correct (out of 6)	4.7	4.7	4.7
Self-reported health status, %			
Excellent/very good	52.3	52.5	53.2
Good	39.9	42.5	38.2
Fair/poor	7.2	5.0	8.7
Total yearly household income, %			
$25,000–$49,999	32.4	29.4	34.3
$50,000–$99,999	35.0	33.4	35.8
$100,000+	31.2	36.3	29.1
Occupation description, %			
Administration/clerical	18.6	11.9	22.9
Customer service/sales	24.2	28.1	21.7
Financial/technical	31.0	35.0	28.5
Management	25.9	25.0	26.5
Mean body mass index, kg/m^2^	31.9	32.8	31.3
Mean systolic blood pressure, mm Hg	118	118	118
Mean diastolic blood pressure, mm Hg	75	77	74
Mean total cholesterol, mg/dl	189	188	189
Mean high density lipoprotein, mg/dl	57	54	59
Mean low density lipoprotein, mg/dl	108	109	107
Mean triglycerides, mg/dl	124	131	120
Mean fasting blood glucose, mg/dl	93	95	92

^a^2 or 3 correct out of 3 items

In terms of biometric data, the mean body mass index in this cohort at baseline is 31.9 kg/m^2^ and does not differ significantly between the intervention and control groups (Table 1). Baseline mean blood pressure, cholesterol levels and fasting glucose levels are similar between groups.

### Calorie purchases during baseline year

During the baseline year, a total of 4721 lunch photographs were submitted from 371 of the enrolled participants (Table 2). The average meal purchased at cafeteria A was 623 (SD 180) calories, and the average meal purchased at cafeterias B and C was 573 (SD 166) calories (Table 2).

**Table 2:** Calories Purchased Baseline Year

	Entire Cohort (*n* = 371)	Cafeteria A (*n* = 146)	Cafeterias B&C (*n* = 225)	*p*-value
Photographs submitted	4721	1891	2830	
Mean calories/meal total	593	623	573	0.007
Standard deviation	173	180	166	

### Physical activity during baseline year

Accelerometers were worn by participants during two separate weeks of the baseline year. There were no significant differences between groups. Among employees utilizing cafeteria A, the average MVPA was 18.9 ± 14.1 min, and among those utilizing cafeterias B and C, the average MVPA was 20.4 ± 14.3 min (Table 3). Based on the weekly assessment, approximately 32% of participants overall were meeting recommended physical activity standard of 150 min of activity per week.

**Table 3:** Physical activity during baseline year

	Entire Cohort (*n* = 262)	Cafeteria A (*n* = 102)	Cafeterias B&C (n = 160)	*P*-value
MVPA^a﻿^ (SD)	19.8 (±14.3)	18.9 (±14.1)	20.4 (±14.3)	0.41
Sedentary minutes (SD)	551 (±69.9)	553 (±74.4)	550 (±66.8)	0.74
Meeting physical activity recommendations^b^ (%)	32.1	31.4	32.5	0.85
Physical activity groups (%)				0.99
Not active	19.5	19.6	19.4	
Some activity	48.5	49.0	48.2	
Active	24.4	24.5	24.4	

^a^MVPA, minutes of moderate or vigorous physical activity

^b^At least 150 min of physical activity

## Discussion

Based on previous research, we know that food labeling with only calorie information is unlikely to be sufficient to motivate healthy eating behavior change. The PACE Study was designed to examine whether a labeling strategy that conveys calorie information in a more readily interpretable format would lead to either a change in calories purchased or in exercise behavior. Strengths of our study include its cohort design, use of meal photographs and a detailed food atlas to assess calorie information, and use of accelerometry to track physical activity data as an outcome. As a cafeteria study, however, its findings may not generalize to other settings such as fast food restaurants.

A reason that calorie information alone may be insufficient to motivate behavior change is that individuals may not understand what calories mean and how the calorie content of an individual item fits into their daily caloric intake, or even what their average daily caloric intake should be [16, 17]. Framing this calorie, or energy intake, by indicating the amount of physical activity required to burn these calories may increase its influence on consumer behavior [4, 8, 16]. According to behavioral economics theory, people will default to using mental shortcuts for many common decisions because our ability to process information is limited [18, 19]. In most cases, particularly when consuming meals not prepared in the home, people’s food-related decisions are not a function of rational processes. Several factors are particularly at odds with the idea that people can rely on a rational, reflective, or cognitive process when making a food choice. The restaurant and fast food environments are designed to capitalize on peoples’ bounded willpower where the “sinful good” of tasty food is not viewed in terms of its potential long-term consequences [18].

Additionally, time pressures are often present, especially over limited food breaks during which many others may be in line to order food. Approaches that rely on cognitive processes are therefore not likely to be effective. In other words, more information is not necessarily what is needed to guide people toward a healthier decision. Approaches that appeal to the intuitive system are much more likely to be effective. Moreover, the appeal needs to happen rather quickly (to compete with time pressure and distractions), which means it needs to be easily understood and interpreted. Thaler and Sunstein popularized the term nudge (in their book by the same name) to describe this type of guiding [20]. In the Health Belief Model, such nudges serve as cues-to-action to motivate behavior change [21]. We hypothesize PACE labeling will be a better “nudge” than calorie-only labeling when considering food purchases.

A study conducted in a laboratory setting found that a convenience sample of participants preferred calorie information with an interpretation aid such as recommended calories per meal, or recommended daily calorie intake, over the number of minutes of running that would be required to burn the calories in that item [17]. Critiques of the physical activity labels included that they had limited generalizability since many people are not able to run, it was discouraging, and most people interpreted the label as a recommendation for exercise rather than a decision making tool that could be used to compare items under consideration [17]. Our PACE label depicts a walking figure as opposed to a running one, which we learned in our formative work was viewed favorably [11].

A recently published study quantified people’s estimation of calories in the meals that they ordered [22]. Among 1161 adults eating a meal in fast food restaurants such as McDonald’s, Burger King, Wendy’s, or Subway in the New England area, the mean actual calorie content of meals purchased was 836 cal. People underestimated the calories in the meals by 175 cal on average, and approximately 25% underestimated calorie content by at least 500 cal. The authors evaluated factors associated with underestimating calories. Of relevance to our work, noticing posted calorie information in the restaurant had no effect on the accuracy of calorie estimations of what people ordered.

We look forward to presenting the results of our post-intervention analyses in future papers. The rigor of our study will advance understanding of food labeling schemes and their potential to help people with their food decisions and exercise behaviors. It will be important to conduct similar studies in different food-ordering environments, especially fast-food establishments.

## Abbreviations

BCBSNC: Blue Cross Blue Shield of North Carolina
CHOW: Capturing Healthy Options at Work
MVPA: Moderate or vigorous physical activity
PACE: Physical Activity Calorie Expenditure
